# Study on causes of fever in primary healthcare center uncovers pathogens of public health concern in Madagascar

**DOI:** 10.1371/journal.pntd.0006642

**Published:** 2018-07-16

**Authors:** Julia Guillebaud, Barivola Bernardson, Tsiry Hasina Randriambolamanantsoa, Laurence Randrianasolo, Jane Léa Randriamampionona, Cesare Augusto Marino, Voahangy Rasolofo, Milijaona Randrianarivelojosia, Ines Vigan-Womas, Voula Stivaktas, Marietjie Venter, Patrice Piola, Jean-Michel Héraud

**Affiliations:** 1 Virology Unit, Institut Pasteur de Madagascar 101, Antananarivo, Madagascar; 2 Epidemiology Unit, Institut Pasteur de Madagascar 101, Antananarivo, Madagascar; 3 Direction de la Veille Sanitaire et de la Surveillance Epidémiologique, Ministry of Public Health 101, Antananarivo, Madagascar; 4 Mycobacterial Unit, Institut Pasteur de Madagascar 101, Antananarivo, Madagascar; 5 Malaria Research Unit, Institut Pasteur de Madagascar 101, Antananarivo, Madagascar; 6 Immunology of Infectious Diseases Unit, Institut Pasteur de Madagascar 101, Antananarivo, Madagascar; 7 Emerging and Respiratory Virus Program, Centre for Viral Zoonoses, University of Pretoria, Pretoria, South Africa; London School of Hygiene&Tropical Medicine, UNITED KINGDOM

## Abstract

**Background:**

The increasing use of malaria diagnostic tests reveals a growing proportion of patients with fever but no malaria. Clinicians and health care workers in low-income countries have few tests to diagnose causes of fever other than malaria although several diseases share common symptoms. We propose here to assess etiologies of fever in Madagascar to ultimately improve management of febrile cases.

**Methodology:**

Consenting febrile outpatients aged 6 months and older were recruited in 21 selected sentinel sites throughout Madagascar from April 2014 to September 2015. Standard clinical examinations were performed, and blood and upper respiratory specimens were taken for rapid diagnostic tests and molecular assays for 36 pathogens of interest for Madagascar in terms of public health, regardless of clinical status.

**Principal findings:**

A total of 682 febrile patients were enrolled. We detected at least one pathogen in 40.5% (276/682) of patients and 6.2% (42/682) with co-infections. Among all tested patients, 26.5% (181/682) had at least one viral infection, 17.0% (116/682) had malaria and 1.0% (7/682) presented a bacterial or a mycobacterial infection. None or very few of the highly prevalent infectious agents in Eastern Africa and Asia were detected in this study, such as zoonotic bacteria or arboviral infections.

**Conclusions:**

These results raise questions about etiologies of fever in Malagasy communities. Nevertheless, we noted that viral infections and malaria still represent a significant proportion of causes of febrile illnesses. Interestingly our study allowed the detection of pathogens of public health interest such as Rift Valley Fever Virus but also the first case of laboratory-confirmed leptospirosis infection in Madagascar.

## Author summary

Febrile illnesses have many origins, but infectious agents are the most important cause. Pathogens such as viruses and bacteria can cause various diseases, but associated signs and symptoms are generally non-specific and can overlap between diseases. Treatments of infectious diseases rely on the diagnostic of the causative agent however laboratory capacities are often lacking in resources-limited settings. Thus, health care workers generally follow clinical management guidelines that are rarely supported by information on the prevalence of local infections. The main objective of our study was to describe the common causes of fever in individuals presenting at outpatient healthcare center in Madagascar. Respiratory viruses, malaria and viral infection in the blood were the main infectious agents detected. Implementation of new diagnostic tools such as bedside testing (Point of Care Testing) could help health care workers to improve diagnosis and care of febrile patients. Detection of other pathogens such as hepatitis B virus, human immunodeficiency virus (HIV) or agent responsible for tuberculosis enhances the need for extended prevention, surveillance and detection of these infections. Interestingly, our study reported cases of leptospirosis and Rift Valley Fever virus infection that are pathogens of high public health concern.

## Introduction

Febrile illness is one of the most common causes of consultations especially in developing countries[1]. A large range of infectious agents may be involved but clinical presentations are often non-specific, hence compromising the validity of diagnosis made with only clinical features[2]. The recent widespread use of rapid diagnosis tests (RDTs) for the diagnostic of malaria infection combined with malaria control interventions lead to a decreasing proportion of confirmed malaria illnesses[3]. Clinical management guidelines for febrile illnesses have been developed but are often syndrome-based[4] and clinical diagnosis can rarely be confirmed as clinical laboratory services are markedly lacking in low-income settings[5]. Clinicians frequently encounter non-malarial febrile cases with few paraclinical options to diagnose and treat patients. Several studies in Eastern Africa[6–9] and Asia[10,11] highlighted the importance of non-malarial infections such as bacterial zoonoses or arboviral infections, which could be prevented and correctly treated if biologically confirmed.

Madagascar is a large island located in the South-Western part of the Indian Ocean that presents different bioclimates and a broad spectrum of malaria transmission. Fever cases are under surveillance since 2007 with the implementation by the Ministry of Public Health (MoH) and the Institut Pasteur de Madagascar (IPM) of a Fever Sentinel Surveillance Network (FSSN) composed of 34 primary health care centers throughout the country[12,13]. This network aims at closely monitoring the following epidemic-prone diseases: malaria, influenza-like illnesses (ILI), Dengue-like Syndromes (DLS) and diarrheal syndromes. From 2008 to 2011, this surveillance reveled that fever is responsible for 11.1% of total outpatients; Among recorded febrile cases, malaria accounted for 12.0%, ILI for 20.7%, DLS for 8.7% and diarrheal diseases for 4.7% [14]. However, only malaria is laboratory confirmed using RDTs following national policies.

Little data is available in Madagascar on the prevalence of other non-malarial infections in humans such as leptospirosis, relapsing fever, rickettsia infection and other less prominent infections[15–19]. The primary objective of our study was to identify the common causes of fever in outpatients attending healthcare centers in Madagascar.

## Methods

### Study design

**Fig 1:**
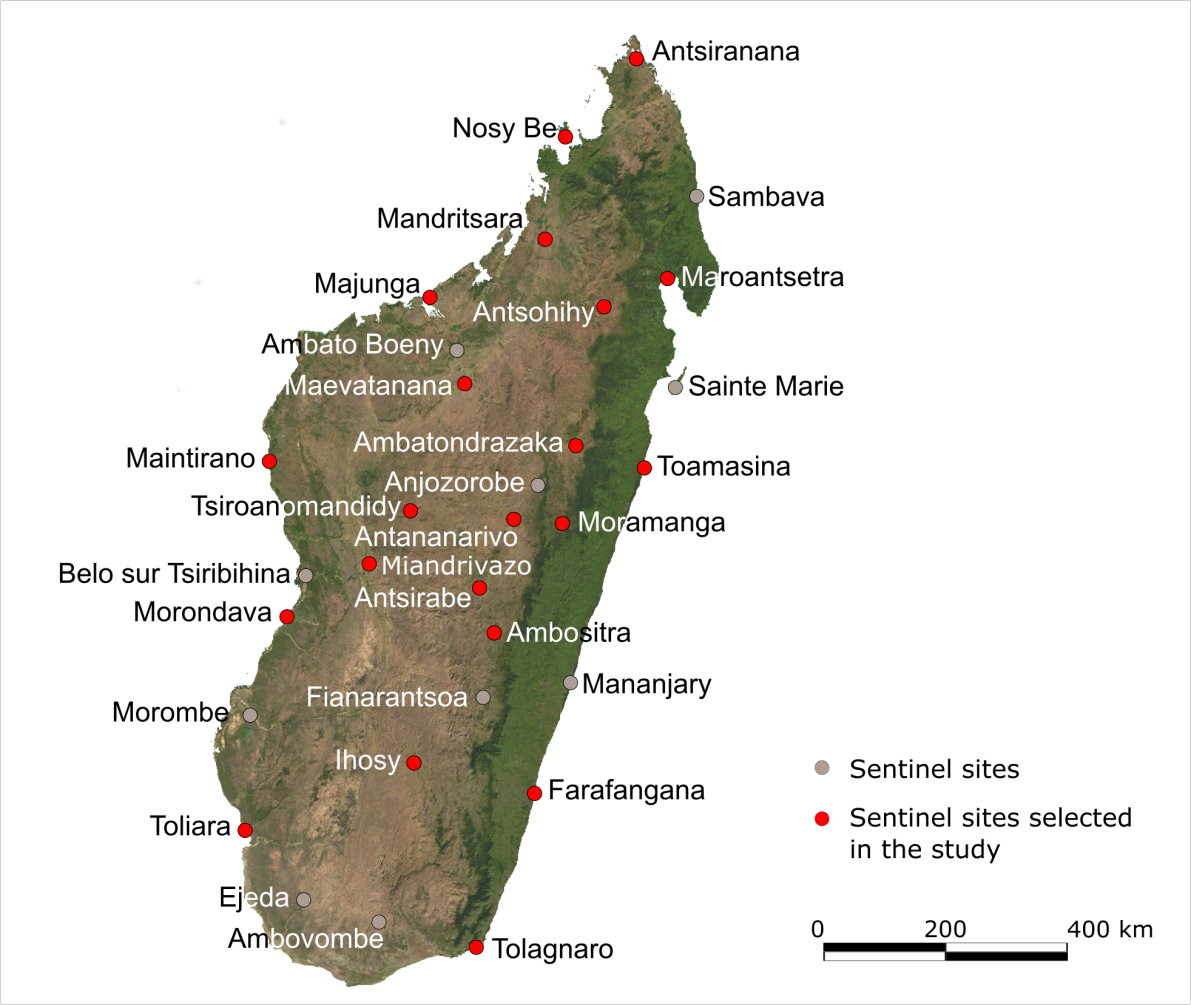
Map of Madagascar showing the 21 sentinel sites selected for the study. [Source: This map was generated using a free source of public domain available at: http://www.maplibrary.org/library/stacks/Africa/Madagascar/index.htm].

A cross-sectional prospective study was conducted in twenty-one sentinel sites (Fig 1) across Madagascar which are representative of the island’s diversity in bioclimates and urban/rural settings. Sites were randomly selected among the 34 sites encompassed in the FSSN. One mobile team from IPM composed of a clinician and a laboratory technician visited all sites during predefined periods between April 2014 and September 2015. We used historical data from the FSSN database to estimate the duration of investigation in each site to reach the number of inclusions. Time of investigation for each site was determined according to various criteria such as availability of the local doctor and team, accessibility of the site and availability of liquid nitrogen. As the objective of our study is to provide a national description of causes of febrile illnesses in a countrywide sentinel network, an overall sample size of 685 consenting patients was deemed adequate to have sufficient precision for prevalent diseases and to detect infrequent pathogens. Hence, the amount of sentinel sites led to a convenience sampling of at least 30 and up to 40 febrile patients per site. A standardized clinical examination was performed and all consenting patients with fever were included and sampled regardless of clinical status.

### Ethics approval and consent to participate

The study protocol was reviewed and approved by the Malagasy National Ethics Committee (approval #CNE 013-MSANP/CE of 26 March 2014). An informed written consent was provided to patients or parents/guardians for children before inclusion and an additional consent form for HIV testing was also provided for every patient included. All samples were anonymized prior to laboratory testing. Two databases were created: one containing all clinical characteristics with names of patients and another one for laboratory results. Both were linked with a unique identifying code for each patient. Individual results of laboratory investigations were returned by mail to health care center clinicians and patients as soon as available.

### Clinical assessment

Each patient attending the study facilities and presenting an uncorrected axillary temperature equal to or exceeding 37.5°C were invited to participate in the study. Exclusion criteria were children aged below 6 months. Written consent was obtained from adult participants and from parents or legal guardians of minors. Additionally, written assent was obtained from patients between 7 and 17 years old. A single anonymous number was attributed to each patient. Voluntary HIV screening (Alere Determine HIV-1/2 according to National HIV Program policies) was also proposed with an additional consent form. Refusal to HIV testing was not an exclusion criterion. Malaria Rapid Diagnostic Test (RDT) (CareStart Malaria HRP2/pLDH (Pf/PAN) Combo, ACCESSBIO) was completed on each febrile patient according to national policies.

A comprehensive clinical examination was performed by the study clinician and a standardized clinical form recorded basic epidemiological and anthropometrical data (S1 Text). A patient with chronic malnutrition was defined as a child below 5 years old with a height-for-age Z-score <-2 Standard Deviation (SD) and a patient with severe acute malnutrition (SAM) as a child below 5 years old with weight-for-height Z-score <-3SD[20]. A total of 49 signs and symptoms were recorded for each participant. Clinical case management of patients was performed by the medical staff of the health center according to national treatment guidelines. Clinical classification of febrile patients was performed according to case definitions used in the FSSN. ILI followed WHO case definition and was defined as a patient presenting a measured axillary temperature equal or exceeding 37.5°C and cough with onset of symptoms within 10 days and not requiring hospitalization[21]. A malaria case was defined as a patient presenting a measured axillary temperature equal or exceeding 37.5°C and a positive malaria RDT (MoH, FSSN). DLS was defined as a patient presenting a measured axillary temperature equal or exceeding 37.5°C and at least two of the following signs: arthralgia, myalgia, headache, rash, retro-orbital pain, hemorrhagic syndrome, in absence of any other suspicious infection–malaria, ILI (MoH, FSSN). Febrile diarrhea followed WHO case definition and was defined as a patient presenting a measured axillary temperature equal or exceeding 37.5°C and presenting the passage of three or more loose or liquid stools per day[22].

### Sampling and laboratory investigations

Nasopharyngeal and/or throats swabs were performed for all patients using Copan swabs and Universal Transport Medium (Copan Diagnostics) in order to detect four respiratory viruses known for their significant prevalence in acute respiratory infections in Madagascar[23]: Influenza A (IAV), Influenza B (IBV), Rhinovirus (HRV) and Respiratory Syncytial Virus (RSV). Molecular techniques were used following the National Influenza Center’s procedures using protocol from the U.S Centers for Disease Control and Prevention (CDC) for influenza typing (Influenza A/B Typing Kit) and an in-house real time RT-PCR duplex for HRV and RSV detection[23].

Patients with productive cough and able to produce sputum had an expectoration sample for tuberculosis screening. Sputum slides were prepared in the field according to the Tuberculosis National Laboratory and stored at room temperature until microscopic examination at the Mycobacteria Unit of IPM; culture of expectorations was performed on Löwenstein-Jensen medium using standard methods back at IPM.

Dried blood spot (DBS) were performed for each patient to perform molecular analysis for malaria species identification using the Malaria Research Unit procedures[24,25].

A whole blood sample (EDTA) was taken to investigate bloodstream infections. When venous sampling was impossible (children below 3 years old, patients refusing venous blood sampling), capillary blood samples were performed using microtube and capillary system (MiniCollect EDTA, Greiner Bio-One). A multiplex PCR-based macroarray assay (Chipron, GmBH), developed and validated at the Emerging vector-borne and respiratory virus program, Centre for Viral Zoonoses, University of Pretoria, South Africa, was used for simultaneous detection of 30 pathogens (S1 Table). The methods previously described[26] combined conventional multiplex PCR with hybridization of biotinylated PCR products to streptavidin labeled probes on a macroarray chip (Chipron GmBH), followed by colorimetric detection. Briefly, each specimen is subjected to 2 multiplex PCR reactions containing biotinylated primers targeted to the 30 pathogens. Subsequently, the PCR products are denatured and hybridized to the chip surface which is coated with target probes directed against each of the 30 pathogens. A positive reaction is detected by addition of streptavidin-conjugated enzyme and substrate, resulting in the development of a color precipitate.

All samples were stored in an electrical cooler at +4°C and were aliquoted and stored in liquid nitrogen within 24h until lab testing. Upper respiratory samples, DBS, sputum slides and samples were tested upon arrival at the central laboratory at IPM. Whole blood samples were first handled for RNA/DNA extraction at the central lab in Madagascar before shipment to the University of Pretoria for grouped analysis.

### Data management and statistical analysis

Data were entered in two Microsoft Access databases (Microsoft Corp, Va., USA) for patient’s data and laboratory findings and linked by the single identification number. Statistical analyses were performed using StataIC 13.1 (Statacorp, College Station, TX). Chi-squared tests were used to compare proportions and categorical variables among groups. Values of p<0.05 were considered significant. Bivariate analysis was performed on all symptoms and syndromes recorded against laboratory findings; symptoms and/or syndromes with a p-value below 0.2 were assessed in a logistic regression and symptoms and/or symptoms with significant OR were retained.

## Results

### Patient’s characteristics and clinical diagnoses

A total of 685 febrile patients were recruited, and only 682 were included for analysis because 3 had insufficient volume of blood collected. Logistic constraints in the site of Maroantsetra have prevented us from reaching the objective of inclusion thus only 28 febrile patients were included. Table 1 presents number of febrile patients included per site and age group. A total of 346 (50.7%) female and 336 (49.3%) male was recruited (F:M ratio = 1.0). Age of patients ranged from 0.5 to 68 years old (mean = 13.5 years, median = 8 years). Patients under 15 years represented 63.0% (429/682) of total inclusions. There was an interval up to 2 days between onset of symptoms and consultation for 59.4% of patients (405/682). The median interval was 2 days, and the mean was 4 days. The median temperature recorded was 38.1°C ranging from 37.5°C to 40.6°C (mean = 38.3°C) with no significant difference between age group and site of inclusion.

**Table 1:** Number of inclusions per site and age group.

Sites of investigation	N	Age group									
		<5 y.		5–14 y.		15–24 y.		25–49 y.		≥50 y.	
Antananarivo	42	14	*33%*	10	*24%*	7	*17%*	9	*21%*	2	*5%*
Farafangana	39	13	*33%*	10	*26%*	10	*26%*	5	*13%*	1	*2%*
Maintirano	41	16	*39%*	10	*24%*	6	*15%*	7	*17%*	2	*5%*
Nosy Be	42	10	*24%*	7	*17%*	10	*24%*	13	*31%*	2	*4%*
Ihosy	41	24	*58%*	6	*15%*	4	*10%*	6	*15%*	1	*2%*
Maroantsetra	28	7	*25%*	10	*36%*	5	*18%*	5	*18%*	1	*3%*
Ambatondrazaka	30	10	*33%*	10	*33%*	5	*17%*	3	*10%*	2	*7%*
Toamasina	30	6	*20%*	4	*13%*	7	*24%*	9	*30%*	4	*13%*
Mahajanga	30	13	*43%*	9	*30%*	5	*17%*	2	*7%*	1	*3%*
Maevatanana	30	13	*43%*	8	*27%*	5	*17%*	3	*10%*	1	*3%*
Antsiranana	30	14	*46%*	9	*30%*	3	*10%*	2	*7%*	2	*7%*
Tsiroanomandidy	30	13	*43%*	4	*13%*	11	*37%*	2	*7%*	0	*0%*
Ambositra	30	11	*37%*	11	*37%*	4	*13%*	4	*13%*	0	*0%*
Morondava	30	22	*74%*	7	*23%*	0	*0%*	1	*3%*	0	*0%*
Miandrivazo	30	15	*50%*	6	*20%*	5	*17%*	4	*13%*	0	*0%*
Mandritsara	30	7	*23%*	11	*37%*	8	*27%*	3	*10%*	1	*3%*
Antsohihy	30	14	*47%*	9	*30%*	4	*13%*	3	*10%*	0	*%*
Toliara	30	12	*40%*	2	*7%*	8	*27%*	7	*23%*	1	*3%*
Sambava	30	7	*23%*	8	*27%*	9	*30%*	6	*20%*	0	*0%*
Taolagnaro	30	15	*50%*	6	*20%*	2	*7%*	7	*23%*	0	*0%*
Moramanga	29	11	*38%*	7	*24%*	4	*14%*	7	*24%*	0	*0%*
**Total**	**682**	**266**	***39%***	**163**	***24%***	**124**	***18%***	**108**	***16%***	**21**	***3%***

Among children below 5 years, the prevalence of chronic malnutrition was 41.7% (111/266) with a wide variation depending on sites (0% to 72.7%)Severe acute malnutrition was present in 6.8% (18/266) of under-fives reaching 23.1% in the site of Farafangana. The S2 Table gives further information on chronic and severe acute malnutrition among enrolled children under 5 years per site.

**Fig 2:**
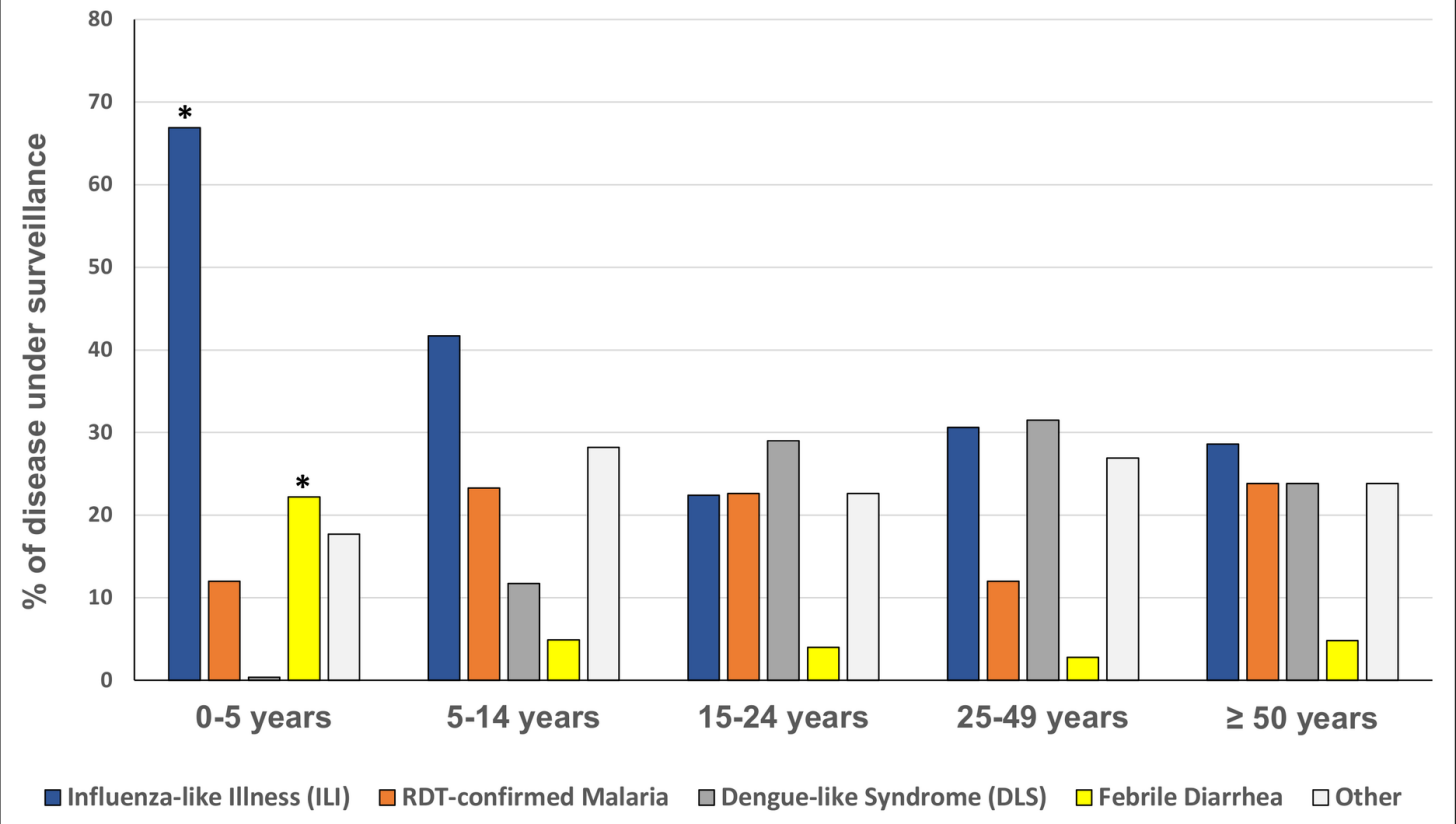
Proportions of diseases under surveillance within the FSSN according to their respective case definition. *p-value <0.05.

Amongst all recorded symptoms and syndromes, we reported 360 cases with headaches (52.8%), 359 cases presenting asthenia (52.6%), 348 cases with catarrh (51.0%), 336 cases with cough (49.3%) and 311 cases presenting anorexia (45.7%). According to case definitions used in the FSSN, 46.8% (319/682) of patients presented an ILI, 17.0% (116/682) had RDT-confirmed malaria, 13.9% (95/682) presented a DLS, and 11.1% (76/682) had febrile diarrhea (Fig 2). ILI and diarrhea cases were significantly more prevalent in children below 5 (p<0.001) with respective proportions of 66.9% and 22.2%. Dengue-like Syndromes were more prevalent in patients older than 5 years (22.6%; p<0.001).

### Laboratory findings and clinical characteristics

Among the 36 pathogens tested, at least one was found in 40.5% (276/682) (Table 2). This proportion varies per age groups from 27.8% (30/108) in adults 25 to 49 years old to 44.4% (118/266) in children below 5 years of age. Positivity rates of the most prevalent pathogens were 17.0% (116/682) for malaria, 8.7% (59/682) for rhinoviruses, 8.4% (57/682) for both A and B influenza viruses, 6.5% (44/682) for Epstein-Barr virus and 3.7% (25/682) for respiratory syncytial viruses.

**Table 2:** Distribution of groups of infection and pathogens detected among febrile patients (N = 682).

Pathogens	Overall n = 682 (%)	<5 years n = 266 (%)	5–14 years n = 163 (%)	15–24 years n = 124 (%)	25–49 years n = 108 (%)	≥50 n = 21 (%)
No pathogen detected	406 (59.5)	148 (55.6)	91 (55.8)	76 (61.3)	78 (72.2)	13 (61.9)
Malaria (RDT)	116 (17.0)	32 (12.0)	38 (23.3)	28 (22.6)	13 (12.0)	5 (23.8)
Virus infections^1^	181 (26.5)	97 (36.5)	42 (25.8)	21 (16.9)	17 (15.8)	4 (19.1)
HRV	59 (8.7)	31 (11.7)*	12 (7.4)	11 (8.9)	5 (4.6)	-
IAV/IBV	57 (8.4)	18 (6.8)	21 (12.9)	11 (8.9)	6 (5.6)	1 (4.8)
EBV	44 (6.5)	29 (10.9)*	6 (3.7)	1 (0.8)	4 (3.7)	4 (19.1)
RSV	26 (3.8)	22 (8.3)*	4 (2.5)	-	-	-
HBV	6 (0.9)	1 (0.4)	2 (1.2)	1 (0.8)	2 (1.9)	-
AdV	2 (0.3)	2 (0.8)	-	-	-	-
CMV	1 (0.2)	1 (0.4)	-	-	-	-
HAV	1 (0.2)	-	-	-	1 (0.9)	-
RVFV	1 (0.2)	-	1 (0.6)	-	-	-
VZV	1 (0.2)	1 (0.4)	-	-	-	-
HIV^3^	1 (0.2)^3^	-	-	-	1 (1.1)^3^	-
Bacterial infections	7 (1.0)	1 (0.4)	1 (0.6)	1 (0.8)	3 (2.8)	1 (4.8)
*M*. *tuberculosis*^2^	4 (0.6)	-	-	-	3 (2.8)	1 (4.8)
*Leptospira spp*.	1 (0.2)	-	-	1 (0.8)	-	-
*N*. *meningitidis*	2 (0.3)	1 (0.4)	1 (0.6)	-	-	-

Abbreviations: RDT = Rapid diagnostic Test; HRV = Human Rhinoviruses; IAV/IBV = Influenza viruses A and B; EBV = Epstein-Barr virus; RSV = Respiratory Syncytial Virus; HBV = Hepatitis B virus; ADV = Adenovirus; CMV = Cytomegalovirus; HAV = Hepatitis A virus; RVFV = Rift Valley Fever virus; VZV = Varicella Zoster virus; HIV = Human Immunodeficiency virus.

^1^ HRV, IAV/IVB and RSV were detected from respiratory specimens. EBV, HBV, ADV, CMV, HAV, RVFV and VZV were detected from blood specimens.

^2^ We only tested patient presenting with cough (n = 23) assuming that patients without cough are non-tuberculosis cases.

^3^ Due to unavailability of rapid test for HIV at the beginning of the study, patients in Antananarivo couldn’t be screened for HIV; A total of 15 patients refused HIV screening. Thus we screened 625 patients including 91 adults aged 25–49 years for HIV and this number was taken as denominator in the calculation of the positivity rate.

* p-value<0.05

Malaria prevalence using RDT varied with sites of investigation from 0.0% in Antananarivo and Moramanga up to 53.3% in Antsohihy. Malaria positivity rate differed per age groups with 12.0% (32/266) in below 5 years, 23.3% (38/163) in 5 to 14 years, 22.6% (28/124) in 15 to 24 years, 12.0% (13/108) in 25 to 49 years and 23.8% (5/21) in above 50 years old (S3 Table). Patients aged 5 years, and more were at higher risk of being infected with malaria compared to children less than 5 years old (p = 0.006).

Molecular tests could be performed on 98.8% (674/682) of DBS. *P*. *falciparum* was detected in 17.1% (115/674) of specimens, *P*. *vivax* in 0.9% (6/674) and *P*. *malariae* in 0.1% (1/674). Among them, three cases of co-infection with *P*. *falciparum* and *P*. *vivax* were identified. Compared to PCR, malaria RDTs sensitivity was 91.6% [CI95: 85.1–95.9] and specificity was 98.8% [CI95: 97.5–99.5].

Amongst the 682 upper respiratory tract specimens, 20.2% (138/682) tested positive for at least one respiratory virus. Influenza A and B represented respectively 4.4% (30/682) and 4.0% (27/682) of infection. Rhinovirus (HRV) and Respiratory Syncytial Virus were respectively detected in 8.4% (59/682) and 3.8% (26/682) of respiratory specimens. Both were significantly more detected in children less than 5 years (p-values of <0.001 with RSV and 0.03 with HRV).

Overall respiratory viruses were more likely to be detected in children below 5 (p = 0.002). Epstein-Barr virus (EBV) was detected in 6.5% (44/682) of patients and significantly more in children below 5 years (29/44) (p<0.001); among them 27.3% (12/44) were co-infected with *Plasmodium*. In total, we screened 625 (91.6%) patients for HIV. One female patient tested positive and was confirmed with subsequent tests according to national guidelines. A total of 23 sputa were collected in patients with productive cough and able to produce sputum. The overall positivity rate for *Mycobacterium tuberculosis* infection was 0.6% (4/682) but is higher at 17.4% (4/23) when considering only patients able to produce sputum. Hepatitis A (HAV) and B (HBV) were detected respectively in 0.1% (1/682) and 0.9% (6/682) of patients.

Among HBV positive patients, three were children below 5 years, two were adolescents (13 and 15 years old) and two were adults (33 and 40 years old). All children presented different abdominal pain (peri umbilical pain, gastralgia and suprapubic pain) and tested positive for malaria, and thus treated accordingly. Adenovirus (ADV) and *Neisseria meningitidis* were each found in 0.3% (2/682) of patients. Cytomegalovirus (CMV), Varicella Zoster virus (VZV) and *Leptospira spp* were detected in 0.1% (1/682) each. Leptospirosis infection was detected in a patient aged 17 years presenting with a high fever (39.7°C) associated with headaches, asthenia, myalgia, chills, nausea, anorexia, malaise, dark urines and tachycardia, and clinically diagnosed with cholecystitis. Leptospirosis infection was subsequently confirmed with PCR[27] and melting temperature superimposition indicated that the strain was related to *L*. *kirshnerii* and *L*. *noguchii* strains. One case of infection with Rift Valley Fever virus (RVFV) was also identified in a 7 years old patient clinically diagnosed with mumps and confirmed by sequencing.

Co-infections represented 6.2% (42/682) of fever and 15.2% of infections (42/276), of which 90.5% (38/42) with 2 pathogens and 9.5% (4/42) with 3 pathogens. Patients infected with at least one virus accounted for 26.5% (181/682). Among the 46.8% (319/682) patients who presented an ILI, 29.8% (95/319) presented a respiratory virus infection with the following pathogens: 13.8% (44/319) influenza virus (A or B), 9.7% (31/319) HRV and 7.5% (24/319) RSV. Three patients were co-infected with IAV and HRV and one with HRV and RSV. Otherwise 8.2% (26/319) had positive malaria RDT, 7.2% (23/319) were infected with EBV, 0.9% (3/319) with *M*. *tuberculosis*, 0.6% (2/319) with ADV, 0.3% (1/319) with CMV, 0.3% (1/319) with VZV and 0.3% (1/319) with *N*. *meningitidis*. Among the 13.9% (95/682) who presented a DLS, no arboviral infection was detected. However, 10.5% (10/95) had a respiratory virus infection with the following pathogens: 6.3% (6/95) with IAV and IBV, and 4.2% (4/95) with HRV. 3.2% (3/95) of patients presented an infection with EBV, 1.1% (1/95) with HBV and 1.1% (1/95) with *Leptospira spp*. Among the 11.1% (76/682) presenting with febrile diarrhea, 22.4% (17/76) had a respiratory virus infection as followed: 11.8% (9/76) with HRV, 7.9% (6/76) with RSV and 2.6% (2/76) with influenza virus (A and B). 9.2% (7/76) presented an EBV infection, 4.0% (3/76) had malaria, 1.3% (1/76) had CMV infection and 1.3% (1/76) had VZV infection. The Table 3 presents results of detection per syndrome/disease under surveillance in the FSSN.

**Table 3:** Distribution of infection detected per syndrome/disease under surveillance within the Fever Sentinel Surveillance Network.

	Fever Sentinel Surveillance Network				
	ILI (n = 319)	Malaria (n = 116)	DLS (n = 95)	Diarrhea (n = 76)	Other (n = 155)
**IAV/IBV**	95 (29.8%)	1 (0.9%)	6 (6.9%)	2 (2.6%)	6 (3.4%)
**HRV**	31 (9.7%)	12 (10.3%)	4 (4.2%)	9 (11.8%)	12 (7.7%)
**RSV**	24 (7.5%)	2 (1.7%)	-	6 (7.9%)	-
**Malaria**	26 (8.2%)	-	-	3 (4.0%)	-
**EBV**	23 (7.2%)	12 (10.3%)	3 (3.2%)	7 (9.2%)	5 (3.2%)
**HBV**	-	4 (3.4%)	1 (1.1%)	-	-
**HAV**	-	-	-	-	1 (0.6%)
**ADV**	2 (0.6%)	-	-	-	-
**CMV**	1 (0.3%)	-	-	1 (1.3%)	-
**VZV**	1 (0.3%)	-	-	1 (1.3%)	-
**RVFV**	-	-	-	-	1 (0.6%)
**HIV**	-	-	-	-	1 (0.6%)
***N*. *meningitidis***	-	-	-	-	1 (0.6%
***Leptospira spp***	-	-	1 (1.1%)	-	-
***M*. *tuberculosis***	3 (0.9%)	-	-	-	1 (0.6%)

Abbreviation: IAV/IBV = Influenza viruses A and B; HRV = Human Rhinoviruses; RSV = Respiratory Syncytial Virus; EBV = Epstein-Barr virus; HBV = Hepatitis B virus; HAV = Hepatitis A virus; ADV = Adenovirus; CMV = Cytomegalovirus; VZV = Varicella Zoster virus; RVFV = Rift Valley Fever virus; HIV = Human Immunodeficiency virus.

Logistic regression of all symptoms and syndromes showed that clinical anemia (OR: 4.9; [CI95: 2.3–10.3]), vomiting (OR: 3.3; [CI95: 2.0–5.4]), headaches (OR: 3.1; [CI95: 1.8–5.6]), chills (OR: 2.5; [CI95: 1.5–4.2]), and sweat (OR: 2.0; [CI95: 1.1–3.6]) were statistically associated with RDT confirmed malaria. Similarly, catarrh, cough, headache and conjunctivitis were statistically associated to Influenza infection with respective OR of 8.2 [CI95: 3.3–20.3], 2.7 [CI95: 1.3–5.4], 2.5 [CI95: 1.2–5.0] and 2.0 [CI95: 1.1–3.7] (S4 and S5 Tables). In our study, WHO ILI case definition[21] had a sensitivity of 77% and a specificity of 56%. Among all patients positive for Influenza virus, 22.8% (13/57) did not meet the WHO ILI case definition as they did not have cough.

## Discussion

Our study aimed at identifying pathogens associated to febrile illnesses in Madagascar. To our knowledge, few studies on etiologies of fever have targeted outpatients’ febrile diseases etiologies widely in a country[10,11], as most focused on severe illnesses and/or children[6,28,29]. Due to non-specificity of some infections and the varieties of pathogens that may circulate concomitantly in tropical countries like Madagascar, laboratory confirmation is needed to estimate the prevalence of each of the pathogens associated to febrile illnesses in the community. We detected at least one pathogen in almost half of the febrile outpatients attending our 21 sentinel sites throughout the country. In this study, ILI was the leading cause of consultation, which is in line with our laboratory results as respiratory viruses were the predominant pathogens detected. Due to financial constraints, we only tested four of these viruses that were found as the most prevalent in previous studies[23,30]. Thus, our detection rate (20.2%) may represent an underestimate in light that other common respiratory viruses such as parainfluenza viruses, human metapneumovirus and coronaviruses might have been detected. This result is consistent with other studies[8,10] where proportions of respiratory viruses detected in nasopharyngeal swabs varied between 11% and 20% and where acute respiratory infections were the major cause of healthcare seeking behavior. In our study, two thirds of patients were under 15 years of age thus explaining the high prevalence of acute respiratory infections[31,32]. Malaria was the second etiology detected followed by viral infection detected in the blood. One patient tested positive for HIV and had a clinical presentation of low grade fever, asthenia, mild cough, lymphadenopathy and leucorrhea. This patient previously tested positive for HIV but had been lost to follow-up on treatment. This situation highlights the need to strengthen screening, care, treatment and follow-up of HIV infections in Madagascar, despite the low prevalence of the infection in the country compared to neighboring African countries[33]. We found a prevalence of active Tuberculosis (TB) infection of 0.6% which is higher that estimated prevalence of tuberculosis in Madagascar (237 per 100 000 populations per year in 2016)[34]. This could emphasize the underestimation of TB diagnosis in health care centers. In addition, TB in children is often missed due to non-specific symptoms and difficulties in diagnosis, thus our results might also underestimate the number of TB cases since we were only able to test patients with productive cough and able to produce sputum. We used predominantly molecular assays to detect pathogens in specimens, allowing high sensitivity of detection although carriage may not necessarily be related to the clinical event[35]. A comparison group of healthy individuals would have allowed us to describe the background level of carriage in the asymptomatic population and estimate the probability that a detected pathogen is truly responsible of the illness. To detect potential multiple infections, we performed the same analyses on all patients regardless of their clinical status. We are aware that this approach cannot be used in an operational manner, but some infrequent pathogens would have been missed if tests had been guided by clinical characteristics. Thus, it allows us to obtain a deeper profile of febrile-associated pathogens. But more importantly, our study allowed us to detect one case of infection with *Leptospira spp*. To our knowledge, this is the first leptospirosis laboratory-confirmed case detected in Madagascar, in Maroantsetra, a city located in a per humid area of the eastern coast. To date, all studies regarding leptospirosis in Madagascar are related to its reservoir or based on serological surveys[15,17]. Only three cases of human leptospirosis infection have already been described since 1955 and only one case of molecular confirmation has been described in La Réunion in a patient with an history of travel to Madagascar[15,36]. As described in previous leptospirosis case reports, this patient presented a suspicion of cholecystitis[37,38]. Interestingly, no bacterial zoonoses diseases like Q fever or rickettsiosis described in other studies to be largely incriminated in fever cases[6,39] were detected, nor arboviral infections. None of the 13.9% of Dengue-like Syndromes were confirmed with dengue virus or other common arboviruses tested, although investigation by IgM ELISA may detect cases that have passed the viremic phase otherwise missed by molecular tests. This emphasizes the low Positive Predictive Value of the DLS case definition when arboviruses (including dengue and chikungunya viruses) prevalence is low and probably its overall limited specificity. HBV was detected in the blood of six patients (0.9%) of which three were children below 5 years. Madagascar has a high-intermediate level of endemicity for HBV infection[40]. National immunization program introduced HBV vaccine in 2002 as part of the pentavalent vaccine after the age of six weeks. These results emphasize not only the need to improve vaccination coverage and prevention of mother to child transmission but also the necessity of scheduling a vaccine dose at birth to avoid perinatal infections. One case of infection with RVFV was detected in a child with initial mumps clinical diagnosis. Several RVFV epidemics occurred in Madagascar in past years[41,42] but as far as we know, no human case was identified during inter-epidemic periods. Our recent data on malnutrition in children less than 5 years are in line with the Millennium Development Goals (MDG) survey 2012–2013, highlighting the need to continue and expand interventions in Madagascar to tackle these conditions.

This study presents some limitations. Indeed, due to resource constraint, some specimens like urine and stools were not collected and we also did not perform hemoculture from blood specimen. Thus, some pathogens associated to sepsis, enteric and urinary tract infections were not investigated. Despite the high sensitivity and specificity of the assay used to detected blood stage infections (Venter et al. 2014), some blood pathogens (such as *Coxiella burnetii* or rickettsial infections) are present at very low levels and nearly impossible to detect in whole blood. Blood cultures and antibodies tests must be used. This might partially explain the percentage of febrile patients with no pathogen detected. The cross-sectional design of our study did not allow assessment of temporal variation of pathogens circulation within the country or a specific area. Given the seasonal pattern of some pathogens (respiratory viruses, malaria, other vector borne diseases) and the design of our study (we visited each site once in a year), we have probably underestimated and/or overestimated some of these pathogens. This is the reason why a comparison of pathogens detected per region is not feasible. Moreover, we believe that the grouping of sites per region or bioclimatic area would not be applicable in this study design. Indeed, socio-economic factors or local transmission pattern of some infectious agents (i.e.: tuberculosis, malaria) would impose separate analysis. Further study should be considered to assess seasonality of fever cases and infectious agents, especially in Madagascar where various bioclimatic regions are encountered. Another limitation is due to the inclusion of mainly patients aged below 15 years overestimating pathogens prevalent in children. However, this age imbalance reflects the true demographics of patients attending health care in Madagascar, where socioeconomic status, travel distance to the facilities and care utilization largely influence healthcare-seeking behavior[43,44]. Nevertheless, the results of our study draw an overview of most prevalent pathogens associated with fever in the country and provide relevant information for further investigations.

Our study contributed to an inventory of pathogens associated to febrile illnesses in Madagascar. Respiratory viruses and malaria are the major causes of febrile illnesses in outpatients. We detected one case of leptospirosis infection and one case of Rift Valley Fever virus infection highlighting a probable circulation of these two pathogens at low level in certain regions of Madagascar since no outbreaks or cases have been reported for these two diseases at the time of our study. Moreover, detections of pathogens such as HAV, HBV and HIV emphasize the need to increase prevention, surveillance, detection and support of these infections. The results of this operational research could help health authorities to prioritize sensitization programs for major febrile illnesses and lay the foundation for relevant Point of Care systems implementation to improve diagnosis and care of febrile patients in Madagascar.

## Supporting information

S1 Text Standardized clinical form used during the study. (DOCX)

S1 Table List of pathogens tested by the macroarray assay developed and validated at the emerging vector borne and respiratory virus program, Centre for Viral Zoonoses, University of Pretoria, South Africa. (DOCX)

S2 Table Chronic and severe acute malnutrition among enrolled children under 5 years per site (n = 266). (DOCX)

S3 Table Distribution of RDT-confirmed malaria patients (RDT+) among febrile patients (n) per site and age groups. (DOCX)

S4 Table Logistic regression table of symptoms explored for RDT-confirmed malaria patients (RDT+). (DOCX)

S5 Table Logistic regression table of symptoms/syndromes explored for influenza-confirmed patients (IAV/IBV). (DOCX)
