# Supplementary material for: Study on causes of fever in primary healthcare center uncovers pathogens of public health concern in Madagascar
Source: PLoS Negl Trop Dis. 2018 Jul 16;12(7):e0006642. doi: 10.1371/journal.pntd.0006642 (PMC6062140; doi:10.1371/journal.pntd.0006642)
Supplement: S1 Text — (DOCX) [file pntd.0006642.s001.docx]

**Criteria for inclusion: any axillary temperature greater than or equal to 37,5°C**

Date: ____ /_____/__________/ Name of the doctor of the clinical study:

**1. Date:** |__||__|/|__||__|/|__||__||__||__| **2.** **Centre:**

**3. District number:** |__||__||__|  **4.** **Name of the doctor of the centre:**

**5*. Patient identification number:* |__||__||__|-|__||__|__|** *i.e.: 001-CUA*

**6. N° Surveillance form (if sentinel site):** |__||__||__||__||__||__||__|

**7. Informed consent signed:** YES □ NO □

**8. Surname:** **9.** **First name:**

**10. Commune: 11. Fokontany:**

**12.** **Sex:** Male □ Female □

**13. Date of birth:** |__||__|/|__||__|/|__||__||__||__|

**14.** **Age :** |__||__| **years** (if age ≤ 2 years, age in months) |__||__| **months**

**15. Fever ≥ 37,5°C :** YES □ NO □ **16. Temperature :** |__||__|, |__| **°C**

**17.** **Date of fever onset:** |__||__|/|__||__|/|__||__||__||__|

**18. Anthropometric parameters:**

| **18.1** | **Weight** | \|__\|\|__\|. \|__\| **kg** |
| --- | --- | --- |
| **18.2** | **Size** | \|__\|\|__\|\|__\|. \|__\| **cm** |
| **18.3** | **MUAC**  *(children less than 5 years old)* | \|__\|\|__\|\|__\| **mm** |

**19. Other similar case(s) in:** the family □ the fokontany □ the village □ Other □

**20. Have you travelled in the 15 days prior to the onset of the disease?** YES □ NO □

**If yes, please specify the place:**

**21. Samples:**

| **Malaria RDT** | YES □ NO □ | Pl. falci □ Other Pl. □ Negative □ |
| --- | --- | --- |
| **Dry blood spot** | YES □ NO □ |  |
| **Blood (dry tube)** | YES □ NO □ |  |
| **Whole blood (EDTA)** | YES □ NO □ |  |
| **Throat swab** | YES □ NO □ |  |
| **Nasopharyngeal swab** | YES □ NO □ |  |
| **Capillary blood collection** | YES □ NO □ | *(Children less than 5 years)* |
| **Sputum** | YES □ NO □ | *(patients with cough and able to produce sputum)* |

**Diagnosis of the attending physician and medical prescription:**

*N° Registry of external consultation:* |__||__||__|

***Patient identification number:* |__||__||__|-|__||__|__|** *i.e.: 001-CUA*

| **Headache** | \|__\| [0-4] | **Allergic syndrome** | \|__\| [0-4] |
| --- | --- | --- | --- |
| **Asthenia** | \|__\| [0-4] | **Herpes labialis** | \|__\| [0-4] |
| **Myalgia** | \|__\| [0-4] | **Erythema** | \|__\| [0-4] |
| **Arthralgia** | \|__\| [0-4] | **Dysentery** | \|__\| [0-4] |
| **Cough** | \|__\| [0-4] | **Dark urines** | \|__\| [0-4] |
| **Catarrh** | \|__\| [0-4] | **Tremors** | \|__\| [0-4] |
| **Dyspnoea** | \|__\| [0-4] | **Skin rash** | \|__\| [0-4] |
| **Sore throat** | \|__\| [0-4] | **Icterus** | \|__\| [0-4] |
| **Retro-orbital pain** | \|__\| [0-4] | **Clinical anaemia** | \|__\| [0-4] |
| **Chills** | \|__\| [0-4] | **Splenomegaly** | \|__\| [0-4] |
| **Vomiting** | \|__\| [0-4] | **Hepatomegaly** | \|__\| [0-4] |
| **Nausea** | \|__\| [0-4] | **Paraesthesia** | \|__\| [0-4] |
| **Anorexia** | \|__\| [0-4] | **Adenopathy** | \|__\| [0-4] |
| **Dizziness** | \|__\| [0-4] | **Haemoptysis** | \|__\| [0-4] |
| **Malaise** | \|__\| [0-4] | **Haematuria** | \|__\| [0-4] |
| **Neck stiffness** | \|__\| [0-4] | **Oliguria** | \|__\| [0-4] |
| **Sweat** | \|__\| [0-4] | **Dehydration** | \|__\| [0-4] |
| **Emaciation** | \|__\| [0-4] | **Photophobia** | \|__\| [0-4] |
| **Conjunctivitis** | \|__\| [0-4] | **Meningeal syndrome** | \|__\| [0-4] |
| **Chest pain** | \|__\| [0-4] | **High blood pressure** | **Value:**  ______/______ |
| **Abdominal pain** | \|__\| [0-4] | **Tachycardia YES □ NO □** | **Pulse:** _______/rpm |
| **Convulsions** | \|__\| [0-4] | **Other(s)** | |
| **Loss of consciousness** | \|__\| [0-4] |  |  |

*[0-4] = Symptom score*

| **Buboes:** | YES □ NO □ | |  |
| --- | --- | --- | --- |
| **Groin** □ **Armpit** □ **Neck** □ **Other location (specify)** □ | | | |
| **Haemorrhagic manifestations:** YES □ NO □ | | | |
| **Purpura** | | | □ |
| **Epistaxis** | | | □ |
| **Gingivorrhagia** | | | □ |
| **Uterine bleeding** | | | □ |
| **Oedema:** YES □ NO □ | | | |
| **Lower limbs** | | | Unilateral □ Bilateral □ |
| **Upper limbs** | | | Unilateral □ Bilateral □ |
| **Face** | | | □ |
| **Other(s)**  (ascite, anasarca…) | | | YES □ NO □ **Specify :** |
| **Lymphangitis:** YES □ NO □ | | | |
| **Lower limbs** | | | Unilateral □ Bilateral □ |
| **Upper limbs** | | | Unilateral □ Bilateral □ |
| **Diarrhoea:** YES □ NO □ | | | |
| **Number of stools issued per 24h:** | | | \|__\|\|__\| |
| **Type of stool:** | | **liquids** | □ |
| **Mucoid** | | | □ |
|  |  |  |  |
| **bloody** | | | □ |
