# Supplementary material for: Study on causes of fever in primary healthcare center uncovers pathogens of public health concern in Madagascar
Source: PLoS Negl Trop Dis. 2018 Jul 16;12(7):e0006642. doi: 10.1371/journal.pntd.0006642 (PMC6062140; doi:10.1371/journal.pntd.0006642)
Supplement: S1 Table — (DOCX) [file pntd.0006642.s002.docx]

**S1 Table:** List of pathogens tested by the macro-array assay developed and validated at the Emerging vector borne and respiratory virus program, Centre for Viral Zoonoses, University of Pretoria, South Africa.

| **Virus** | **Bacteria** | **Haemoparasite** |
| --- | --- | --- |
| West Nile Virus | *Rickettsia spp* | *Plasmodium falciparum* |
| Rift Valley Fever Virus | *Borrelia burgdorferi* |  |
| Chikungunya Virus | *Brucella spp* |  |
| Sindbis Virus | *Coxiella burnetii* |  |
| Rubella Virus | *Leptospira spp* |  |
| Crimean Congo Haemorragic Fever Virus | *Mycobacterium tuberculosis* |  |
| Cytomegalo Virus | *Ehrlichia spp* |  |
| Measle Virus | *Nesseiria meningitidis* |  |
| Mumps Virus |  |  |
| Herpes simplex Virus type 1 |  |  |
| Herpes simplex Virus type 2 |  |  |
| Varicella Zoster Virus |  |  |
| Rabies Virus |  |  |
| Epstein Barr Virus |  |  |
| JC-Virus |  |  |
| Enterovirus |  |  |
| Dengue Virus |  |  |
| Flavivirus genre I & II |  |  |
| Hepatitis A Virus |  |  |
| Hepatitis B Virus |  |  |
| Adenovirus |  |  |
