# Supplementary material for: Study on causes of fever in primary healthcare center uncovers pathogens of public health concern in Madagascar
Source: PLoS Negl Trop Dis. 2018 Jul 16;12(7):e0006642. doi: 10.1371/journal.pntd.0006642 (PMC6062140; doi:10.1371/journal.pntd.0006642)
Supplement: S2 Table — (DOCX) [file pntd.0006642.s003.docx]

**S2 Table:** Chronic and severe acute malnutrition among enrolled children under 5 years per site (n=266).

| **Sites** | **N** | **Chronic malnutrition (%)** | **Severe acute malnutrition (%)** |
| --- | --- | --- | --- |
| Antananarivo | 14 | 6 (42.9) | 1 (7.2) |
| Farafangana | 13 | 3 (23.1) | 3 (23.1) |
| Maintirano | 16 | 5 (31.3) | 3 (18.8) |
| Nosy Be | 10 | 3 (30.0) | 0 (0.0) |
| Ihosy | 24 | 13 (54.2) | 2 (8.3) |
| Maroantsetra | 7 | 0 (0.0) | 0 (0.0) |
| Ambatondrazaka | 10 | 5 (50.0) | 0 (0.0) |
| Toamasina | 6 | 1 (16.7) | 0 (0.0) |
| Mahajanga | 13 | 6 (46.2) | 2 (15.4) |
| Maevatanana | 13 | 8 (61.5) | 1 (7.7) |
| Antsiranana | 14 | 4 (28.6) | 2 (14.3) |
| Tsiroanomandidy | 13 | 6 (46.2) | 0 (0.0) |
| Ambositra | 11 | 8 (72.7) | 0 (0.0) |
| Morondava | 22 | 14 (63.6) | 1 (4.6) |
| Miandrivazo | 15 | 6 (40.0) | 2 (13.3) |
| Antsohihy | 14 | 4 (28.6) | 0 (0.0) |
| Mandritsara | 7 | 2 (28.6) | 0 (0.0) |
| Toliara | 11 | 4 (36.4) | 0 (0.0) |
| Sambava | 7 | 0 (0.0) | 0 (0.0) |
| Taolagnaro | 15 | 9 (60.0) | 0 (0.0) |
| Moramanga | 11 | 4 (36.4) | 1 (9.1) |
| All sites | 266 | 111 (41.7) | 18 (6.8) |
| *Chronic malnutrition was defined as a child below 5 years old with a height-for-age Z-score <-2 Standard Deviation (SD) and severe acute malnutrition as a child below 5 years old with weight-for-height Z-score <-3SD^18^* | | | |
