# Supplementary material for: Study on causes of fever in primary healthcare center uncovers pathogens of public health concern in Madagascar
Source: PLoS Negl Trop Dis. 2018 Jul 16;12(7):e0006642. doi: 10.1371/journal.pntd.0006642 (PMC6062140; doi:10.1371/journal.pntd.0006642)
Supplement: S3 Table — (DOCX) [file pntd.0006642.s004.docx]

**S3 Table:** Distribution of RDT-confirmed malaria patients (RDT+) among febrile patients (n) per site and age groups.

| **Site** | **Overall** | | **<5 years** | | **5-14 years** | | **15-24 years** | | **25-49 years** | | **≥50 years** | |
| --- | --- | --- | --- | --- | --- | --- | --- | --- | --- | --- | --- | --- |
|  | **n** | **RDT+ (%)** | **n** | **RDT+ (%)** | **n** | **RDT+ (%)** | **n** | **RDT+ (%)** | **n** | **RDT+ (%)** | **n** | **RDT+ (%)** |
| **Antananarivo** | 42 | 0 (0.0) | 14 | 0 (0.0) | 9 | 0 (0.0) | 8 | 0 (0.0) | 9 | 0 (0.0) | 2 | 0 (0.0) |
| **Farafangana** | 39 | 20 (51.3) | 13 | 3 (23.1) | 10 | 8 (80.0) | 10 | 6 (60.0) | 5 | 2 (40.0) | 1 | 1 (100) |
| **Maintirano** | 41 | 16 (39.0) | 16 | 5 (31.3) | 10 | 7 (70.0) | 6 | 3 (50,0) | 7 | 1 (14.3) | 2 | 0 (0.0) |
| **Nosy Be** | 42 | 5 (11.9) | 10 | 0 (0.0) | 7 | 1 (14.3) | 10 | 2 (20.0) | 13 | 1 (15.4) | 2 | 0 (0.0) |
| **Ihosy** | 41 | 2 (4.9) | 24 | 1 (4.2) | 6 | 0 (0.0) | 4 | 0 (0.0) | 6 | 0 (0.0) | 1 | 1 (100) |
| **Maroantsetra** | 28 | 2 (7.2) | 7 | 0 (0.0) | 10 | 1 (10.0) | 5 | 0 (0.0) | 5 | 1 (20.0) | 1 | 0 (0.0) |
| **Ambatondrazaka** | 30 | 1 (3.3) | 10 | 0 (0.0) | 10 | 1 (10.0) | 5 | 0 (0.0) | 3 | 0 (0.0) | 2 | 0 (0.0) |
| **Toamasina** | 30 | 0 (0.0) | 6 | 0 (0.0) | 4 | 0 (0.0) | 7 | 0 (0.0) | 9 | 0 (0.0) | 4 | 0 (0.0) |
| **Mahajanga** | 30 | 2 (6.7) | 13 | 1 (7.7) | 9 | 1 (11.1) | 5 | 0 (0.0) | 2 | 0 (0.0) | 1 | 0 (0.0) |
| **Maevatanana** | 30 | 12 (40.0) | 13 | 7 (53.9) | 8 | 1 (12.5) | 5 | 1 (20.0) | 3 | 2 (66.7) | 1 | 1 (100) |
| **Antsiranana** | 30 | 1 (3.3) | 14 | 0 (0.0) | 9 | 0 (0.0) | 3 | 0 (0.0) | 2 | 0 (0.0) | 2 | 1 (50.0) |
| **Tsiroanomandidy** | 30 | 4 (13.3) | 13 | 1 (7.7) | 4 | 1 (25.0) | 11 | 2 (18.2) | 2 | 0 (0.0) | 0 | NA |
| **Ambositra** | 30 | 3 (10.0) | 11 | 0 (0.0) | 11 | 0 (0.0) | 4 | 2 (50.0) | 4 | 1 (25.0) | 0 | NA |
| **Morondava** | 30 | 3 (10.0) | 22 | 0 (0.0) | 7 | 3 (42.9) | 0 | NA | 1 | 0 (0.0) | 0 | NA |
| **Miandrivazo** | 30 | 7 (23.3) | 15 | 2 (13.3) | 6 | 2 (33.3) | 5 | 2 (40.0) | 4 | 1 (25.0) | 0 | NA |
| **Antsohihy** | 30 | 16 (53.3) | 14 | 7 (50.0) | 9 | 6 (66.7) | 4 | 3 (75.0) | 3 | 0 (0.0) | 0 | NA |
| **Mandritsara** | 30 | 6 (20.0) | 7 | 2 (28.6) | 11 | 2 (18.2) | 8 | 1 (12.5) | 3 | 0 (0.0) | 1 | 1 (100) |
| **Toliara** | 30 | 6 (20.0) | 12 | 0 (0.0) | 2 | 0 (0.0) | 8 | 4 (50.0) | 7 | 2 (28.6) | 1 | 0 (0.0) |
| **Sambava** | 30 | 3 (10.0) | 7 | 1 (14.3) | 8 | 1 (12.5) | 9 | 1 (11.1) | 6 | 0 (0.0) | 0 | NA |
| **Taolagnaro** | 30 | 7 (23.3) | 15 | 2 (13.3) | 6 | 3 (50.0) | 2 | 1 (50.0) | 7 | 1 (14.3) | 0 | NA |
| **Moramanga** | 29 | 0 (0.0) | 11 | 0 (0.0) | 7 | 0 (0.0) | 4 | 0 (0.0) | 7 | 0 (0.0) | 0 | NA |
| **All sites** | **682** | **116 (17.0)** | **266** | **32 (12.0)** | **163** | **38 (23.3)** | **124** | **28 (22.6)** | **108** | **13 (12.0)** | **21** | **5 (23.8)** |
| Abbreviation: RDT=Rapid diagnostic Test. NA= Non-applicable (no patient tested). | | | | | | | | | | | | |
