# Supplementary material for: Study on causes of fever in primary healthcare center uncovers pathogens of public health concern in Madagascar
Source: PLoS Negl Trop Dis. 2018 Jul 16;12(7):e0006642. doi: 10.1371/journal.pntd.0006642 (PMC6062140; doi:10.1371/journal.pntd.0006642)
Supplement: S4 Table — (DOCX) [file pntd.0006642.s005.docx]

**S4 Table:** Logistic regression table of symptoms explored for RDT-confirmed malaria patients (RDT+).

|  | **Univariate analysis** | | | | **Multivariate analysis** | | | |
| --- | --- | --- | --- | --- | --- | --- | --- | --- |
| **Symptoms** | **Odd Ratio** | **95% CI** | | ***P value*** | **Odd Ratio** | **95% CI** | | ***P value*** |
|  |  | **Lower** | **Upper** |  |  | **Lower** | **Upper** |  |
| **Headache** | 3.8 | 2.3 | 6.3 | <0.001 | 3.1 | 1.8 | 5.6 | <0.001* |
| **Myalgia** | 2.2 | 1.3 | 3.6 | <0.001 | 1.2 | 0.6 | 2.5 | 0.551 |
| **Arthralgia** | 1.6 | 1.0 | 2.5 | 0.0399 | 0.7 | 0.4 | 1.4 | 0.388 |
| **Chills** | 3.8 | 2.4 | 5.8 | <0.001 | 2.5 | 1.5 | 4.2 | <0.001* |
| **Vomiting** | 2.8 | 1.8 | 4.4 | <0.001 | 3.3 | 2.0 | 5.4 | <0.001* |
| **Nausea** | 1.7 | 1.0 | 2.7 | 0.0237 | 0.7 | 0.4 | 1.3 | 0.259 |
| **Anorexia** | 1.8 | 1.2 | 2.8 | 0.0021 | 1.3 | 0.8 | 2.1 | 0.338 |
| **Vertigo** | 2.2 | 1.4 | 3.4 | <0.001 | 0.9 | 0.5 | 1.5 | 0.632 |
| **Malaise** | 1.7 | 1.1 | 2.8 | 0.0144 | 0.6 | 0.3 | 1.2 | 0.121 |
| **Sweat** | 2.8 | 1.8 | 4.5 | <0.001 | 2.0 | 1.1 | 3.6 | 0.018* |
| **Abdominal pain** | 3.1 | 2.0 | 4.9 | <0.001 | 1.5 | 0.9 | 2.6 | 0.100 |
| **Dark urine** | 5.2 | 1.8 | 15.1 | 0.0011 | 1.5 | 0.3 | 6.8 | 0.594 |
| **Tremors** | 4.8 | 1.8 | 12.7 | <0.001 | 3.0 | 1.0 | 8.5 | 0.043 |
| **Icterus** | 8.5 | 1.6 | 54.9 | 0.0048 | 2.7 | 0.4 | 16.5 | 0.295 |
| **Clinical anemia** | 6.6 | 3.5 | 12.3 | <0.001 | 4.9 | 2.3 | 10.3 | <0.001* |
| **Splenomegaly** | 2.8 | 0.7 | 9.5 | 0.0721 | 2.5 | 0.6 | 10.6 | 0.226 |
| **Hematuria** | 3.9 | 1.4 | 10.3 | 0.0042 | 1.5 | 0.5 | 4.7 | 0.510 |
| **Oliguria** | 2.5 | 0.4 | 11.8 | 0.1852 | 1.7 | 0.3 | 10.6 | 0.586 |
| **Dehydration** | 1.7 | 0.7 | 4.2 | 0.1388 | 0.5 | 0.2 | 1.5 | 0.243 |
| **Photophobia** | 2.5 | 0.8 | 7.4 | 0.0683 | 1.2 | 0.3 | 4.3 | 0.819 |
| *Data are expressed as odd ratio (OR) (95% confidence interval). Logistic regression was conducted with significant variables on bivariate analysis. P value<0.05 is statistically significant and marked with asterisk (*).* | | | | | | | | |
|  |  |  |  |  |  |  |  |  |
|  |  |  |  |  |  |  |  |  |
