# Supplementary material for: Study on causes of fever in primary healthcare center uncovers pathogens of public health concern in Madagascar
Source: PLoS Negl Trop Dis. 2018 Jul 16;12(7):e0006642. doi: 10.1371/journal.pntd.0006642 (PMC6062140; doi:10.1371/journal.pntd.0006642)
Supplement: S5 Table — (DOCX) [file pntd.0006642.s006.docx]

**S5 Table:** Logistic regression table of symptoms/syndromes explored for Influenza-confirmed patients (IAV/IBV).

|  | **Univariate analysis** | | | | **Multivariate analysis** | | | |
| --- | --- | --- | --- | --- | --- | --- | --- | --- |
| **Symptoms** | **Odd Ratio** | **95% CI** | | ***P value*** | **Odd Ratio** | **95% CI** | | ***P value*** |
|  |  | **Lower** | **Upper** |  |  | **Lower** | **Upper** |  |
| **Headache** | 1.5 | 0.8 | 2.7 | 0.1104 | 2.5 | 1.2 | 5.0 | 0.011* |
| **Asthenia** | 1.4 | 0.8 | 2.5 | 0.1664 | 1.4 | 0.8 | 2.6 | 0.275 |
| **Cough** | 3.9 | 2.0 | 8.0 | <0.001 | 2.7 | 1.3 | 5.4 | 0.005* |
| **Catarrh** | 7.8 | 3.5 | 20.8 | <0.001 | 8.2 | 3.3 | 20.3 | <0.001* |
| **Retro-orbital pain** | 1.7 | 0.8 | 3.5 | 0.1075 | 0.9 | 0.4 | 2.3 | 0.851 |
| **Vertigo** | 1.4 | 0.7 | 2.5 | 0.1949 | 1.8 | 0.8 | 3.9 | 0.147 |
| **Conjunctivitis** | 2.9 | 1.6 | 5.3 | <0.001 | 2.0 | 1.1 | 3.7 | 0.030* |
| **Photophobia** | 2.3 | 0.4 | 8.3 | 0.1845 | 4.1 | 0.9 | 18.2 | 0.074 |
| *Data are expressed as odd ratio (OR) (95% confidence interval). Logistic regression was conducted with significant variables on bivariate analysis. P value<0.05 is statistically significant and marked with asterisk (*).* | | | | | | | | |
|  |  |  |  |  |  |  |  |  |
|  |  |  |  |  |  |  |  |  |
